# *S*-Glutathionylation-Controlled Apoptosis of Lung Epithelial Cells; Potential Implications for Lung Fibrosis

**DOI:** 10.3390/antiox11091789

**Published:** 2022-09-10

**Authors:** Elizabeth Corteselli, Reem Aboushousha, Yvonne Janssen-Heininger

**Affiliations:** Department of Pathology and Laboratory of Medicine, University of Vermont Larner College of Medicine, Burlington, VT 05405, USA

**Keywords:** glutaredoxin, glutathione, apoptosis, idiopathic pulmonary fibrosis, epithelial cells

## Abstract

Glutathione (GSH), a major antioxidant in mammalian cells, regulates several vital cellular processes, such as nutrient metabolism, protein synthesis, and immune responses. In addition to its role in antioxidant defense, GSH controls biological processes through its conjugation to reactive protein cysteines in a post-translational modification known as protein *S*-glutathionylation (PSSG). PSSG has recently been implicated in the pathogenesis of multiple diseases including idiopathic pulmonary fibrosis (IPF). Hallmarks of IPF include repeated injury to the alveolar epithelium with aberrant tissue repair, epithelial cell apoptosis and fibroblast resistance to apoptosis, and the accumulation of extracellular matrix and distortion of normal lung architecture. Several studies have linked oxidative stress and PSSG to the development and progression of IPF. Additionally, it has been suggested that the loss of epithelial cell homeostasis and increased apoptosis, accompanied by the release of various metabolites, creates a vicious cycle that aggravates disease progression. In this short review, we highlight some recent studies that link PSSG to epithelial cell apoptosis and highlight the potential implication of metabolites secreted by apoptotic cells.

## 1. Glutathione and *S*-Glutathionylation 

Tripeptide glutathione (GSH) is the most abundant antioxidant in mammalian cells [1]. Glutathione contains the amino acids glutamate, cysteine, and glycine, and exists in reduced (GSH) and oxidized disulfide (GSSG) forms. Glutathione molecules containing additional sulfur atoms and with unique chemical reactivity also exist, but will not be further discussed here [1,2,3,4,5]. Under homeostatic conditions, the ratio of GSH:GSSG heavily favors the former (99:1), reflecting a reducing environment; although, in other compartments such as the endoplasmic reticulum and extracellularly, this ratio is more oxidizing [6,7]. GSH exerts numerous vital functions in cells, including phase II xenobiotic metabolism and reactions with oxidants [1,8,9]. In addition, GSH regulates physiological processes that include cell proliferation [10,11,12], DNA and protein synthesis [13,14], cytokine production, and immune responses [15,16,17]. Through the γ-glutamyl cycle, GSH acts as a continuous source of cysteine that can be incorporated into GSH synthesis itself, or in the synthesis of other proteins [18]. One critical function of GSH that is now well established is its conjugation to protein thiols in a post-translational modification called protein *S*-glutathionylation (PSSG). Multiple protein cysteine oxidations occur, including of sulfenic acid residues (SOH) and *S*-nitrosylated residues (SNO). While SOH- and SNO-containing proteins impart unique biological functions, SOH and SNO moieties can also give rise to more stable PSSG. PSSG changes the structure and function of the protein of concern by affecting the charge and size. Additionally, PSSG is chemically important as it prevents the overoxidation of cysteines [4,19,20,21,22]. The forward *S*-glutathionylation reaction can be catalyzed by glutathione *S*-transferases, notably by GSTP, which is the highest-expressed GST in lung tissue (see [23] for recent review). Conversely, glutaredoxin under physiological conditions acts as a deglutathionylating enzyme re-establishing the reduced protein thiol, as will be further discussed below. Because PSSG is reversible, it facilitates the dynamic regulation of the protein structure and function, making this post-translational modification an ideal mechanism through which signals from oxidants are transduced into biological responses. 

## 2. Alterations in Redox Homeostasis in Idiopathic Pulmonary Fibrosis

The lung is constantly exposed to high oxygen concentrations and has some of the highest GSH levels in the body [24,25,26]. As such, dysregulation of the GSH system has been implicated in diverse lung pathologies, including cystic fibrosis, chronic obstructive pulmonary disease, asthma, and lung fibrosis. Idiopathic pulmonary fibrosis (IPF) is a progressive and irreversible interstitial lung disease characterized by the deposition of the extracellular matrix and loss of lung function. IPF is an aging-associated disease and occurs primarily in older adults, preferentially males, and has increased in both prevalence and mortality in recent years [27]. Patients diagnosed with IPF have a poor prognosis with a median survival of 3-5 years following diagnosis. Relatively few therapeutic options exist, and care is focused on managing symptoms and slowing disease progression [28]. The key histopathological features of IPF include the loss of the alveolar epithelium, the appearance of honeycombed cysts myofibroblasts and fibroblast foci, and the excess deposition of the extracellular matrix in the distal regions of the lung. As its name suggests, the exact cause of IPF is not currently known. However, several processes are thought to be central to IPF pathogenesis, such as repeated cycles of epithelial cell injury and death, aberrant repair along with changes in epithelial cell plasticity, sustained transforming growth factor beta (TGFB) signaling, and fibroblast activation to a myofibroblast phenotype that is accompanied by resistance to apoptosis and senescence [29,30,31].

Changes in redox homeostasis have been observed in patients with IPF and mouse models of fibrosis. Oxidized proteins, hydrogen peroxide, nitric oxide, and 8-isoprostanes, a byproduct of lipid peroxidation, were significantly increased in the bronchoalveolar lavage fluid (BALF) and exhaled breath was found to condensate in patients with IPF compared to healthy controls [32,33,34]. In addition, blood samples from IPF patients showed evidence of increased oxidant formation [35]. Among the intracellular sources of oxidants in cells are the NADPH family of oxidases (NOX), which produce hydrogen peroxide and superoxide [36]. Notably, a prominent role for NOX4 in pulmonary fibrosis was identified [37,38]. NOX4 was increased in the alveolar epithelium of IPF lungs, and global deletion of NOX4 protected mice from bleomycin-induced fibrosis [39]. In a bleomycin mouse model of fibrosis, persistent fibrosis was detected in aged mice compared to young mice and was accompanied by an apoptosis-resistant population of myofibroblasts. Intriguingly, the apoptosis resistance of aged myofibroblasts was mediated by the elevated expression of NOX4, as well as an impaired capacity to induce NRF2, which is a transcription factor that augments the expression of numerous antioxidant and redox-controlling genes. A small-molecule NOX4 inhibitor diminished age-associated persistent bleomycin-induced fibrosis in mice, while NOX4 siRNA diminished senescence and apoptosis resistance in fibroblasts in association with a diminished expression of the antiapoptotic protein BCL2 [40]. Activation of NRF2 was also sufficient to reverse the myofibroblast phenotype [40,41]. These findings illuminate an age-dependent imbalance of the NOX4–NRF2 axis in the bleomycin model of fibrosis in mice. Importantly, concomitant increases in the expression of NOX4 and a diminished expression of NRF2 were also demonstrated in primary fibroblasts isolated from the lungs of IPF subjects, compared to fibroblasts from control subjects or lung tissues [40]. 

Decreases in GSH have also been observed in fibrotic lungs. Sputum samples from patients with IPF showed significantly decreased GSH when compared to healthy controls, with a slight inverse correlation between sputum GSH and disease progression [42]. The same trend was observed in the lower respiratory tract, where epithelial lining fluid from IPF patients also demonstrated a fourfold decrease in GSH [43]. In addition, a decreased GSH [42] and a decreased GSH:GSSG ratio were observed in blood samples from patients with IPF. 

In recent years, it has become evident that fibrosis may not simply result from an imbalance between the production of oxidants and antioxidant defenses, but that an altered redox environment affects sensitive proteins and pathways in a cell-specific and location-specific manner. This specificity of oxidant-induced targets is perhaps evidenced by the failure of broadly applied non-specific antioxidant therapies for fibrosis, such as *N*-acetyl-l-cysteine, which demonstrated no significant benefit in clinical trials [44,45]. These observations indicate that a greater understanding of the oxidative processes and affected cellular pathways in IPF is necessary to provide more promising therapeutic options. With numerous clinical and animal studies confirming a redox imbalance in IPF, some have tried to characterize an oxidative biomarker to predict disease progression and acute exacerbations [46]. However, no consensus about the utility of such biomarker has yet been reached and these endeavors have not yielded a widely utilized assay for clinical management.

## 3. Glutaredoxin and Lung Fibrosis

A key regulator of PSSG is the family of oxidoreductases called glutaredoxins (GLRX). Since the first description of GLRX [47], several isoforms of GLRX have been described and studied. Mammalian GLRX1, hereafter referred to as GLRX, is the main isoform in the cytosol, which can catalyze both the removal of glutathione from a protein thiol (deglutathionylation) and the reduction of disulfide bonds. The first step of the deglutathionylation reaction involves the breaking of a mixed protein-GSH disulfide by GLRX, which occurs rapidly due to the low pKa (approximately 3.5) of the GLRX active site cysteine [48]. As a result of this reaction, GLRX itself is glutathionylated, and must be reduced by a second molecule of GSH. The glutathione system, specifically GSH, Glutathione Reductase (GR), and NADPH, provides reduction equivalents to fuel these reactions [5,49]. 

In mammalian cells, GLRX is the primary enzyme responsible for deglutathionylation in the cytosol. Although they are members of the thioredoxin protein superfamily, GLRX reduces mixed protein-GSH disulfides much faster, and with greater specificity, than thioredoxins [50]. As such, the balance of glutathionylated proteins depends heavily on the expression and activity of GLRX. Mice with a global deletion of *Glrx* appear grossly normal, and do not display signs of increased oxidative stress under homeostatic conditions [51]. However, the consequences of an altered *Glrx* expression become evident in pathophysiological settings in which the *Glrx* status is linked to the aberrant activation of various pathways that control disease progression in diverse tissues. For example, GLRX-deficient mice fed a high-fat diet develop steatohepatitis [52], a condition that can lead to liver fibrosis. In contrast, the upregulation of GLRX enhances neuroinflammation and neuronal cell death, and contributes to the progression of Parkinson’s disease [53]. GLRX has also been implicated in angiogenesis, with increases in GLRX inhibiting vascularization and endothelial cell migration [54]. Interestingly, sexual dimorphism was recently reported for the role of GLRX in metabolic reprogramming of monocytes, where GLRX deficiency in female monocytes promotes dysregulated macrophage phenotypes and weight gain [55]. For more detailed information on GLRX in brain, liver, and heart pathophysiology, the reader is directed to the recent and comprehensive review by Matsui et al. [56]. 

Alterations in GLRX activity and expression have been associated with pulmonary fibrosis. GLRX expression and activity were decreased in patients with IPF compared to healthy controls in association with increases in PSSG. In addition, GLRX activity was directly correlated with lung function, while PSSG was inversely correlated with lung function in subjects with IPF. In models of bleomycin- or TGFB1-induced fibrosis, mice lacking *Glrx* displayed significantly increased collagen deposition, while transgenic mice overexpressing *Glrx* in airway epithelial cells showed attenuated collagen deposition. Strikingly, the oropharyngeal administration of recombinant GLRX was sufficient to reverse existing fibrotic changes [57]. The pathways in which disrupted PSSG and GLRX promote fibrogenesis remain incompletely understood and await elucidation of the *S*-glutathionylated proteome. However, one prominent PSSG target that contributes to the development of lung fibrosis is the death receptor FAS, as is described below. 

## 4. Importance of Apoptosis in Lung Fibrosis

Epithelial cells and fibroblasts are cell types with critical roles in the pathogenesis of pulmonary fibrosis. In fibrotic lung disease, including IPF, disruptions of the distal conducting airway or alveolar epithelial cells represent common histopathological features. Inhaled insults lead to the death of epithelial cells; in an aging lung, the lack of normal epithelial restitution along with the concomitant activation and proliferation of myofibroblasts collectively promote fibrosis [58,59,60,61,62]. Apoptosis is a form of programmed cell death that is crucial for normal homeostasis and is dysregulated in diverse pathologies. It involves the tightly regulated recruitment and binding of specific receptor-ligand pairs to induce activation of proteolytic effector caspases for controlled degradation of cellular components. Two main mechanisms of apoptosis have been described in detail. The first involves the translocation of Bid to the mitochondria, resulting in the release of caspases to the cytosol, commonly referred to as the intrinsic pathway. In contrast, the extrinsic pathway involves the ligand binding of cell surface receptors, including FAS, TNF, D4, and D5, to form a death-induced signaling complex (DISC), which further recruits and activates caspases [63]. 

Numerous studies strongly support the importance of increased epithelial apoptosis, and the apoptosis resistance of myofibroblasts, in subsequent fibrogenesis and the importance of FAS herein. Upon administration of diphtheria toxin to transgenic mice expressing the diphtheria toxin receptor in type II epithelial cells or club cells, marked death of type II epithelial or club cells occurs with resultant prolonged fibrosis [61,62]. The functional role of FAS in the development of pulmonary fibrosis is evident from studies showing that agonistic FAS antibody (which mimics crosslinking between FASL and FAS) resulted in the apoptosis of epithelial cells, leading to fibrosis [58,64,65]. Conversely, bleomycin-induced fibrosis could be prevented using soluble anti-FAS, or anti-FAS Ligand antibodies, and did not occur in mice that lacked functional FAS (*lpr*) or FAS ligand (*gld*) [57,66]. Apoptosis of epithelial cells has been shown following co-culture with myofibroblasts isolated from patients with IPF that express FASL, while these myofibroblasts themselves are resistant to FASL-induced apoptosis [65,67]. The resistance of IPF myofibroblasts to FAS-induced apoptosis has been associated with an increased expression of multiple antiapoptotic genes and the downregulation of FAS [68,69]. The selective ablation of FAS in lung fibroblasts led to fibroblast and fibrosis persistence in mice [69], which confirmed the importance of diminished FAS expression to the apoptosis resistance of myofibroblasts. Epithelial cell death by IPF-derived fibroblasts has been linked to H_2_O_2_ produced by myofibroblasts [70]. Taken together, these findings demonstrate the importance of FAS in epithelial cell apoptosis and resistance to FAS-induced apoptosis in myofibroblasts in the pathogenesis of lung fibrosis. These findings suggest that avenues that dampen the extent of epithelial cell death and promote the apoptosis sensitivity of fibroblasts by targeting the FAS pathway in epithelial cells and fibroblasts, have the potential to attenuate fibrotic remodeling. 

## 5. Glutathionylation and Epithelial Cell Apoptosis

Several studies have noted a link between the glutathione system and cell death by apoptosis. Deletion of the GSH synthesis enzyme GCLC in mice is embryonically lethal due to the high rates of apoptosis [71]. The reduction in cytosolic GSH is a hallmark of cells undergoing apoptosis [72], and GSH depletion through cysteine starvation has been shown to induce apoptosis [73]. Conversely, increased GSH levels are associated with resistance to apoptosis [74,75]. Further, there is evidence that apoptotic proteins can themselves be glutathionylated. Our laboratory has previously shown that the activation of apoptosis in airway epithelial cells induced by FASL is accompanied by increases in total PSSG [76]. In this model, FAS was found to be glutathionylated at cysteine 294 following the binding of FASL, thereby promoting caspase activation and apoptosis. Increases in FAS-SSG were not linked to increased oxidant production, but rather, were linked to the degradation of GLRX by caspases. Epithelial cells lacking *Glrx* were more susceptible to FASL-induced apoptosis and displayed elevated FAS-SSG compared to WT counterparts, while the overexpression of *Glrx* attenuated epithelial cell apoptosis in association with diminished FAS-SSG. A follow-up study conducted by our group discovered the existence of distinct pools of FAS in epithelial cells and that upon ligation of FAS, the latent FAS localized in the endoplasmic reticulum undergoes oxidative processing in a reaction that requires protein disulfide isomerase A3 (PDIA3) and GSTP, leading to the formation of FAS-SSG. Knock down, ablation, or pharmacological inhibition of PDIA3 or GSTP attenuated FAS-SSG and epithelial apoptosis [77]. Of relevance are further studies demonstrating that the pharmacological inhibition of PDIA3 or GSTP using clinically relevant compounds attenuated fibrosis in multiple mouse models [77,78]. Besides FAS, caspase-3 is also regulated via PSSG. In endothelial cells during tumor necrosis factor alpha (TNF-A)-induced apoptosis, caspase-3-SSG prevented its cleavage by caspase-8 and attenuated apoptosis [79]. Control of caspase-3-SSG was GLRX dependent, with the expression of GLRX in turn regulated by TNF-A. Collectively, these observations highlight the importance of the PSSG of key proteins in the apoptosis pathway in regulating the extent of apoptosis.

## 6. Release of Metabolites by Apoptotic Cells: Potential for Regulation by Glutaredoxin 

As described above, pulmonary fibrosis is the culmination of enhanced apoptosis of epithelial cells, an acquired state of apoptosis resistance of myofibroblasts, and aberrant cross talk between epithelial cells, fibroblasts, and likely additional cell types not discussed here. These observations raise questions about whether signals originating from apoptotic epithelial cells impinge on the biology of fibroblasts and other cell types within the lung. A recent study shed light on this question by subjecting a variety of cells to multiple inducers of apoptosis and conducted a metabolite analysis of the medium. They reported that a number of metabolites were consistently released from multiple cell types subjected to various apoptotic stimuli, including the stimulation of FAS. These metabolites included spermidine, creatine, dihydroxyacetone phosphate (DHAP), fructose-1,6-bisphosphate (FBP), UDP-glucose, glycerol-3-phosphate, (G3P), guanosine 5′ monophosphate (GMP), and inosine monophosphate (IMP). The authors also reported that six of these metabolites together profoundly affected gene expression profiles in macrophages and that these effects could be recapitulated by three out of six metabolites, namely spermidine, GMP, and IMP. Importantly, these metabolites were also anti-inflammatory in vivo and elicited protective effects in arthritis and lung transplant rejections in mouse models. The pannexin 1 channel, which opens in a caspase-dependent manner, was required for metabolite release [80].

**Figure 1:**
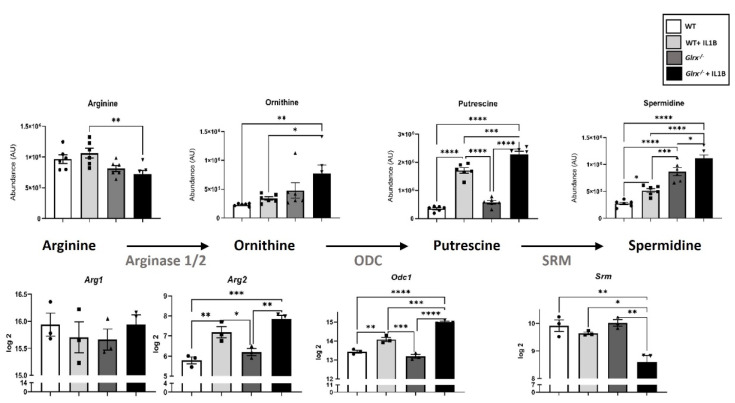
GLRX-dependent modulation of metabolic pathways that use arginine and give rise to spermidine in airway basal cells stimulated with IL1B. WT or *Glrx^−/−^* airway basal cells were stimulated with 10 ng/mL of IL1B for 24 h prior to the assessment of gene expression or metabolite analysis in cell lysates. We refer the reader to Aboushousha et al. [17] for technical details about this study. * *p* < 0.05, ** *p* < 0.01, *** *p* < 0.001, **** *p* < 0.0001, ANOVA.

**Figure 2:**
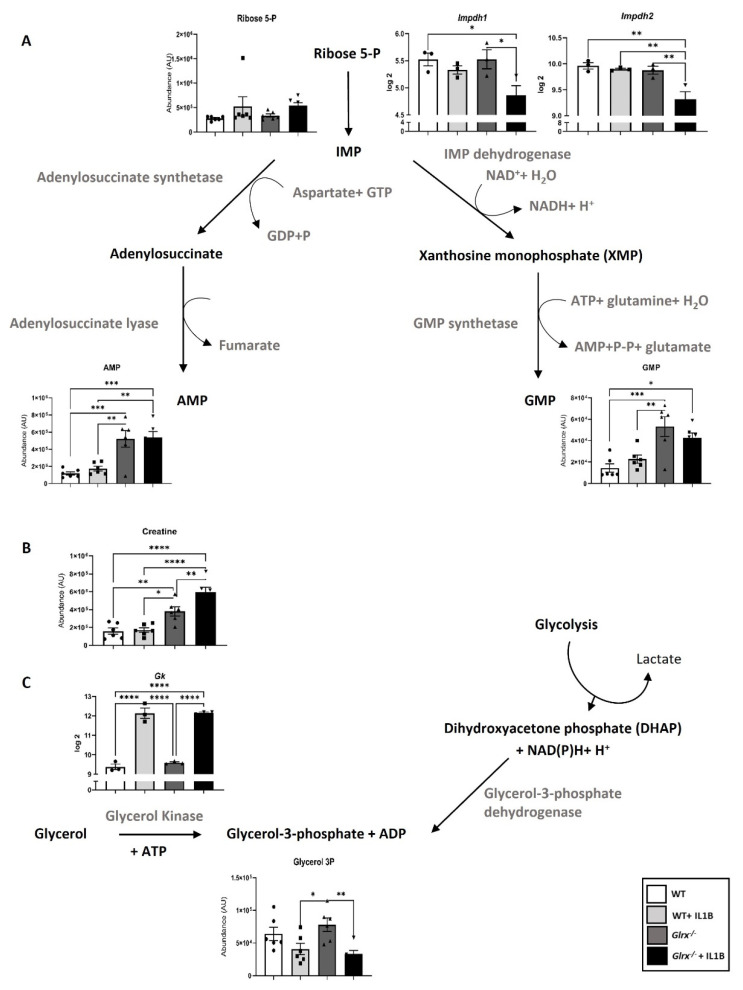
GLRX-dependent modulation of metabolic pathways that regulate AMP, GMP (**A**), creatine (**B**), and glycerol-3-phosphate **(C)** in airway basal cells stimulated with IL1B. WT or *Glrx^−/−^* airway basal cells were stimulated with 10 ng/mL of Il1B for 24 h prior to the assessment of gene expression or metabolite analysis in cell lysates. We refer the reader to Aboushousha et al. [17] for technical details about this study. * *p* < 0.05, ** *p* < 0.01, *** *p* < 0.001, **** *p* < 0.0001, ANOVA.

The identity and importance of metabolites released from apoptotic epithelial cells in fibrotic lung tissues remain unknown, and the importance of oxidative signals in promoting metabolite formation and release also require additional investigation. We conducted a multi-omics study in airway epithelial cells derived from WT or *Glrx^−/−^* mice stimulated with the pro-fibrotic cytokine interleukin 1 beta (IL1B), and discovered that multiple metabolic pathways were perturbed in the absence of *Glrx* or in response to IL1B [17]. Notably, glycolysis was enhanced in epithelial cells lacking *Glrx*, with further increases occurring following IL1B stimulation. When further assessing the metabolite profiles in this study, we observed that some of the metabolites released by apoptotic cells were also detected intracellularly and depended on the GLRX status. As stated above, *Glrx^−/−^* epithelial cells are more prone to FASL-induced apoptosis [76]. The results in Figure 1 show that *Glrx^−/−^* epithelial cells had higher levels of spermidine. Stimulating WT or *Glrx^−/−^* cells with IL1B increased spermidine levels in WT and *Glrx^−/−^* cells, with further increases apparent in *Glrx^−/−^* cells. In mammalian cells, arginine is a major source of spermidine, via the arginase (ARG)-dependent conversion of arginine to ornithine, the subsequent ornithine decarboxylase (ODC)-dependent conversion of ornithine to putrescine, and finally via spermidine synthase (SRM) conversion of putrescine to spermidine (Figure 1). While arginine, ornithine, and putrescine levels were not different between unstimulated WT or *Glrx^−/−^* epithelial cells, in response to IL1B arginine diminished while ornithine and putrescine levels increased in cells lacking *Glrx* compared to their WT counterparts. Supporting these observations, mRNA levels of arginase 2 (*Arg2*) were increased in control or IL1B-stimulated *Glrx^−/−^* cells compared to the respective WT groups. Ornithine decarboxylase (*Odc*) was increased in IL1B-stimulated *Glrx^−/−^* cells compared to the respective WT group. In contrast, spermidine synthase (*Srm*) decreased in *Glrx^−/−^* cells stimulated with IL1B compared to the WT group (Figure 1). Collectively, these findings suggest that a metabolic pathway that yields spermidine is regulated by GLRX status. Among the other metabolites released by apoptotic cells were GMP, AMP, creatine, and G3P [80]. Of interest, *Glrx^−/−^* cells exhibited higher levels of GMP, AMP (Figure 2A), and creatine (Figure 2B), while G3P levels decreased in *Glrx^−/−^* cells following IL1B stimulation (Figure 2C). Inosine monophosphate dehydrogenases 1 and 2 (*Impdh1/2*) catalyze the conversion of ribose-5-phosphate to inosine monophosphate (IMP), which can subsequently be metabolized to xanthosine monophosphate and GMP via Inosine-5′-monophosphate dehydrogenase (IMPDH) and GMP synthase. In response to IL1B, the expression of *Impdh1* and *Impdh2* significantly decreased compared to unstimulated *Glrx^−/−^* cells or the WT groups (Figure 2A). While the expression of arginine:glycine amidinotransferase and guanidinoacetate *N*-methyltransferase, which generate creatine, remained unchanged in our study (not shown), the expression of glycerol kinase (*Gk*), which catalyzes the conversion of glycerol to G3P, significantly increased in response to IL1B in both WT and *Glrx^−/−^* cells (Figure 2C). Overall, these findings illustrate the modulation of multiple pathways that regulate the synthesis and utilization of diverse metabolites released by apoptosis cells and their regulation by GLRX status. 

## 7. Conclusions and Future Directions 

**Figure 3:**
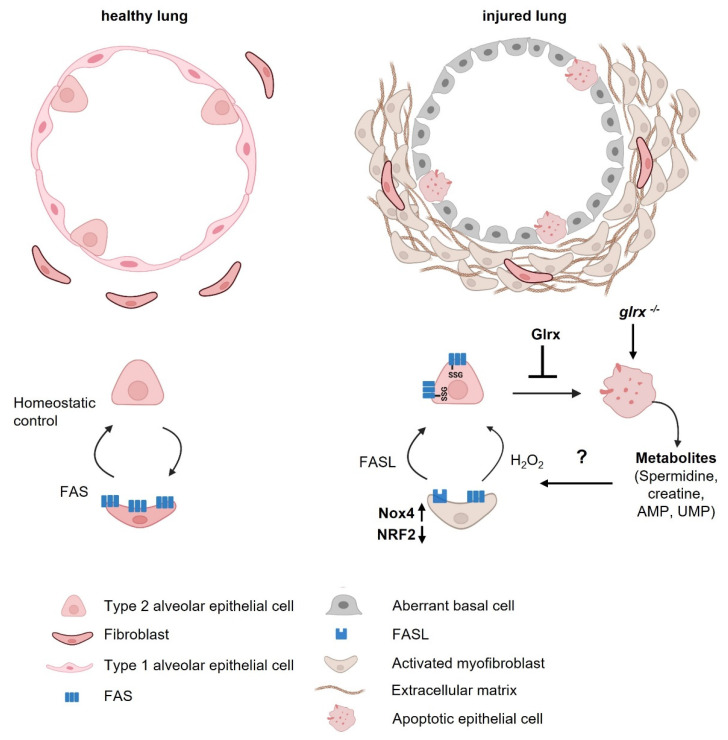
Schematic overview of disrupted epithelial-fibroblast homeostasis in pulmonary fibrosis. Left: top—depiction of the healthy alveolus lined with alveolar type 1 (AT1) cells interspersed with alveolar type 2 (AT2) progenitor cells and sporadic fibroblasts. Bottom—homeostatic control between AT2 cells and fibroblasts. Right: top—depiction of a disrupted alveolus in pulmonary fibrosis showing loss of AT1 cells, dying AT2 cells, emerging aberrant basal cells, activated fibroblasts, and excessive deposition of extracellular matrix. Bottom—disrupted crosstalk between AT2 cells and fibroblasts in fibrosis. Enhanced FASL and NOX4 expression in fibroblasts and secretion of H_2_O_2_ promote killing of alveolar epithelial cells, while the diminished FAS expression on activated myofibroblasts conversely promotes their apoptosis-resistance. Enhanced FAS-SSG on epithelial cells promotes their death, which can be ameliorated by the deglutathionylase, GLRX. Additionally depicted is the GLRX-sensitive production of apoptosis-associated metabolites in dying alveolar epithelial cells. Future studies should address the impact of apoptosis-associated metabolites in the emergence of aberrant basal cells and myofibroblasts and the disrupted cross talk between epithelial cells and fibroblasts. This image was generated using Biorender.

Pulmonary fibrosis is an insidious disease that develops in response to diverse insults against the backdrop of aging. Aberrant death of epithelia, disrupted differentiation patterns of progenitor epithelial cells, emerging populations of apoptosis-resistant myofibroblasts, and the production of an excessive extracellular matrix are some of the contributory processes that promote the loss of normal alveolar architecture. These abnormalities occur in an increased oxidative environment, represented by increases in the oxidation of GSH, enhanced *S*-glutathionylation, and disruptions in GLRX (Figure 3). Remarkably, the exact PSSG targets in different cell populations or the extracellular environment of the fibrotic lung remain unknown. The implications of enhanced PSSG for dying epithelial cells also require further study. In this manuscript, we addressed the importance of GLRX for epithelial cell apoptosis and the production of metabolites in apoptosis-prone epithelial cells. The exact PSSG target(s) that regulates the formation and utilization of these metabolites remain(s) to be formally tested. The implications of elevated levels of these metabolites in *Glrx^−/−^* cells, their secretion, and implications for (myo)fibroblast biology also warrant further investigation. Additional studies will also be required to assess whether other pro-fibrotic stimuli besides IL1B can regulate the aforementioned pathways and metabolites. Of note, a recent study reported that the supplementation of primary human corneal fibroblasts with arginine increased a number of metabolites, including spermidine, and was associated with increases in collagen deposition [81]. Conversely, treatment of mice with spermidine following the development of bleomycin-induced fibrosis caused decreases in alveolar epithelial cell apoptosis, and ultimately decreased inflammation and collagen deposition [82]. An intricate link exists between redox-dependent processes including *S*-glutathionylation, energy metabolism, and cell death, all of which are processes that are disrupted in lung fibrosis. Multi-omics-based approaches that aim at identifying oxidation-sensitive nodes that intersect these pathways in different cell populations disrupted in the fibrotic lung have great potential to shed further light on disease pathogenesis, and may yield therapeutic strategies not realized to date. With the availability of high resolution redox-mapping [83], single cell-based mass spectrometry [84], and organoid technology to expand diseased cell populations [85,86], these studies are now possible and will yield new insights into redox, metabolic, and cellular perturbations with unprecedented detail. 

## Abbreviations

AMP	adenosine monophosphate
AT1	alveolar type 1 cells
AT2	alveolar type 2 cells
BALF	bronchoalveolar lavage
DHAP	dihydroxyacetone phosphate
DISC	death-induced signaling complex
FBP	fructose-1,6-bisphosphate
G3P	glycerol-3-phosphate
GK	glycerol kinase
GLRX	glutaredoxin
GMP	guanosine 5′ monophosphate
GSH	glutathione
GSSG	oxidized glutathione
GSTP	glutathione *S*-transferases P
GR	glutathione reductase
IMP	inosine monophosphate
IMPDH	inosine monophosphate dehydrogenases
IPF	idiopathic pulmonary fibrosis
NOX	NADPH family of oxidases
ODC	ornithine decarboxylase
PDIA3	protein disulfide isomerase A3
PSSG	protein *S*-glutathionylation
SRM	spermidine synthase
TNF-A	tumor necrosis factor alpha
TGFB	transforming growth factor beta
